# Monitoring Long Term Noninvasive Ventilation: Benefits, Caveats and Perspectives

**DOI:** 10.3389/fmed.2022.874523

**Published:** 2022-05-19

**Authors:** Jean-Paul Janssens, Chloé Cantero, Patrick Pasquina, Marjolaine Georges, Claudio Rabec

**Affiliations:** ^1^Division of Pulmonary Diseases, Department of Medicine, Geneva University Hospitals, Geneva, Switzerland; ^2^Hôpital de La Tour, Centre Cardio-Respiratoire, Geneva, Switzerland; ^3^Faculty of Medicine, University of Geneva, Geneva, Switzerland; ^4^Service de Pneumologie, Hôpital Pitié-Salpêtrière AP-HP – Sorbonne Université, Paris, France; ^5^Pulmonary Department and Respiratory Critical Care Unit, University Hospital Dijon, Dijon, France

**Keywords:** non-invasive ventilation, chronic hypercapnic respiratory failure, monitoring, home ventilation, long term mechanical ventilation

## Abstract

Long term noninvasive ventilation (LTNIV) is a recognized treatment for chronic hypercapnic respiratory failure (CHRF). COPD, obesity-hypoventilation syndrome, neuromuscular disorders, various restrictive disorders, and patients with sleep-disordered breathing are the major groups concerned. The purpose of this narrative review is to summarize current knowledge in the field of monitoring during home ventilation. LTNIV improves symptoms related to CHRF, diurnal and nocturnal blood gases, survival, and health-related quality of life. Initially, patients with LTNIV were most often followed through elective short in-hospital stays to ensure patient comfort, correction of daytime blood gases and nocturnal oxygenation, and control of nocturnal respiratory events. Because of the widespread use of LTNIV, elective in-hospital monitoring has become logistically problematic, time consuming, and costly. LTNIV devices presently have a built-in software which records compliance, leaks, tidal volume, minute ventilation, cycles triggered and cycled by the patient and provides detailed pressure and flow curves. Although the engineering behind this information is remarkable, the quality and reliability of certain signals may vary. Interpretation of the curves provided requires a certain level of training. Coupling ventilator software with nocturnal pulse oximetry or transcutaneous capnography performed at the patient's home can however provide important information and allow adjustments of ventilator settings thus potentially avoiding hospital admissions. Strategies have been described to combine different tools for optimal detection of an inefficient ventilation. Recent devices also allow adapting certain parameters at a distance (pressure support, expiratory positive airway pressure, back-up respiratory rate), thus allowing progressive changes in these settings for increased patient comfort and tolerance, and reducing the requirement for in-hospital titration. Because we live in a connected world, analyzing large groups of patients through treatment of “big data” will probably improve our knowledge of clinical pathways of our patients, and factors associated with treatment success or failure, adherence and efficacy. This approach provides a useful add-on to randomized controlled studies and allows generating hypotheses for better management of HMV.

## Introduction

Long term noninvasive ventilation (LTNIV) is widely accepted for treatment of chronic hypercapnic respiratory failure (CHRF) related to obstructive or restrictive disorders. Its development was triggered in the early 1980's by the report by Sullivan et al. of the use of CPAP for obstructive sleep apnea syndrome (1). Since then devices have undergone major evolutions, with a progressive shift from volume-cycled to pressure-cycled then multimodal devices, and appearance of auto-titrating modes which can adapt to changes in airway resistance, reactance, or compliance of the respiratory system and aim to ensure a target tidal volume and minute ventilation (2). One of the major contributions of medical engineering for these devices has been the development of built-in software, allowing to monitor in detail items such as compliance, leaks, tidal volume, residual respiratory events, or percentage of cycles triggered/cycled by the device (3–7). Moreover, for some devices, analysis of raw data for flow, pressure and even thoraco-abdominal movements on a breath-by-breath basis is possible. Strategies for monitoring LTNIV have also evolved: historically, elective evaluations of patients on LTNIV were initially most often hospital-based or even ICU-based; at present, they rely more and more on tools such as pulse oximetry, transcutaneous capnography (PtcCO_2_) and built-in software that can be assessed at home (4). New developments in telemedicine and the growing contribution of health care providers make these data easily available, allowing to remotely assess quality of ventilation.

In this review, we will discuss the benefits, but also the possible pitfalls of monitoring patients under LTNIV, and perspectives as to future options. This review will focus on an adult population under LTNIV.

### Why Monitor LTNIV?

The goals of long-term NIV (LTNIV) are to improve symptoms related to chronic hypercapnia and—when present—sleep-related breathing disorders (SRBD), to improve health-related quality of life (HRQL), decrease hospital admissions, correct hypoventilation and optimize SpO_2_ in order to prevent the development of pulmonary hypertension and cor pulmonale, and improve survival (8). In several indications (chronic obstructive pulmonary diseases (COPD), amyotrophic lateral sclerosis (ALS), restrictive thoracic disorders), improvement of ABG and control of nocturnal hypoventilation are significantly associated with a better prognosis (9–13). In order to achieve these goals, it is mandatory to ensure that: 1/ LTNIV improves daytime and nocturnal arterial blood gases, 2/ compliance to LTNIV is sufficient to have a significant physiological effect, 3/ LTNIV does not generate *per se* undesired respiratory events or side effects and discomfort, and 4/ HRQL and symptom scores improve over time. Guidelines usually recommend at least one annual elective assessment for patients under LTNIV: the frequency of evaluations depends however on the underlying pathology, its severity and rate of progression, and may be required as often as every 3 months (8, 14, 15).

### Monitoring LTNIV: A Step by Step Approach

#### Daytime and Nocturnal Blood Gases: Capillary, Transcutaneous or Arterial Samples

Normalizing daytime arterial blood gases (ABG) is a major physiological goal for patients under LTNIV (9, 10). Thus, performing daytime measurements of ABG—by puncture of the radial artery—is a standard procedure when monitoring LTNIV. Measurements are performed without NIV, at least 30 min after interrupting NIV (when possible), preferably in a sitting position. Capillary arterialized samples, taken at the earlobe after application of a vasodilating gel, are considered reliable surrogates of arterial ABG for pH and PaCO_2_ (16–19). Results for PaO_2_ are less reliable, with a possible underestimation by the capillary technique (20).

In stable chronic respiratory failure, pH is usually within a normal range (2, 21–24). The contribution of bicarbonates to the detection of residual nocturnal hypoventilation is debated because of the impact of a large panel of drugs and comorbidities on HCO_3_ levels (25, 26). HCO_3_ levels should not be used as an isolated criterion for detecting nocturnal hypoventilation, but can be indicative of nocturnal hypoventilation and should lead to nocturnal capnography if available.

Daytime ABG measurements under NIV are indicative of correction of PaCO_2_ by NIV but have been shown to be a poor reflection of correction of nocturnal hypoventilation (27–29). Thus, assessing nocturnal pCO_2_ is essential for identifying periods of hypoventilation related to leaks, undesired respiratory events including patient-ventilator asynchrony or inappropriate settings.

Nocturnal arterial punctures are a source of discomfort, awaken the patient, and are not recommended. Surrogates of nocturnal ABG assessment are end-tidal CO_2_ (ETCO_2_) and transcutaneous capnography (TcPCO_2_). Reliability of ETCO_2_ measurements is subject to V/Q mismatch, physiological dead space and ventilatory mode. Importantly, a normal ETCO_2_ does not exclude hypercapnia, while an elevated ETCO_2_ is strongly suggestive of hypercapnia but may underestimate actual PaCO_2_ value (4, 30). Thus, the use of ETCO_2_ as a surrogate measurement for nocturnal PaCO_2_ is not recommended in most circumstances (4).

Transcutaneous capnography (TcPCO_2_) is considered reliable for continuous (nocturnal) monitoring of PaCO_2_ when patients are hemodynamically stable, and devices are used by an experienced team. A probe temperature of 42°C is tolerated for 8 h without any risk of skin burns in adults (31). Several studies have reviewed the performances of commercialized capnographs and show acceptable biases and limits of agreement (27, 29). A review of the recent literature on transcutaneous capnography suggested that bias values up to ± 1 kPa (7.5 mmHg), and limits of agreement up to ± 1.33 kPa (10 mmHg) were acceptable (27). PtcCO_2_ however has a few drawbacks: 1/ its lag time which precludes the detection of very short events (32) and 2/ the occurrence of occasional errand values, largely dependent on the expertise of the users.

Consensual—although arbitrary—definitions for hypoventilation have been published by the American Association for Sleep Medicine (33). However, minor changes in definitions of hypoventilation can have a major impact on clinical assessment (34–36).

Pulse oximetry has the advantage of simplicity and low cost: its major drawback is the lack of specificity of desaturations, which do not allow to distinguish hypoventilation from other causes of drops in SpO_2_.

### Contribution of Built-In Monitoring Devices

#### Compliance

**Figure 1:**
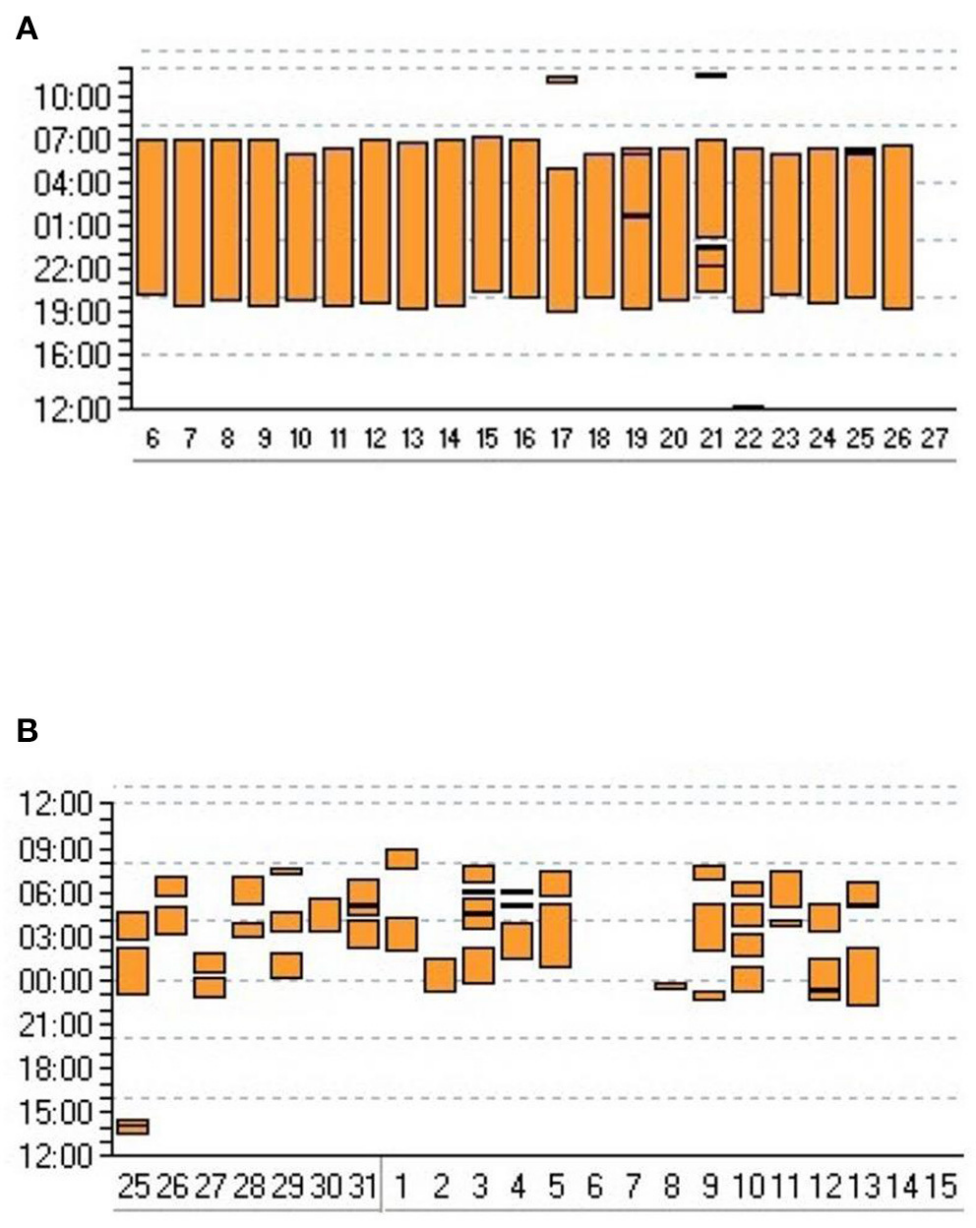
Adherence to NIV. Graphic transcription of ventilator use provided by ventilator software. Y axis: time of the day/night; X axis: days. Each vertical bar represents time spent using the ventilator for a given day. **(A)** Very regular use suggesting that patient is adherent and comfortable with his/her treatment. **(B)** Frequent interruptions and irregular use of ventilator suggesting discomfort and/or comorbidity.

Compliance is directly related to efficacy of NIV (on ABG and symptom control), and prognosis; it may also reflect patient discomfort, which impairs health related quality of life. Analysis of use of LTNIV (compliance) has 3 components: total time spent with the ventilator, pattern of use and evolution of these parameters (Figure 1). Total time spent under NIV has to be long enough for NIV to have a beneficial impact on daytime and nocturnal ABG, and, in some cases, on total energy expenditure (37, 38). It is usually considered that using NIV <3:30 h per 24 h is insufficient and does not allow resetting of respiratory centers and resting of respiratory muscles. Borel et al. described a “U-shaped curve” relationship between compliance and prognosis (39). Too low values provide insufficient benefit, while higher values indicate more severe respiratory impairment (39). In COPD, a meta-analysis by Struik et al. suggests that a daily use of NIV for at least 5 h is required to improve PaCO_2_ (40). In neuromuscular disorders such as amyotrophic lateral sclerosis or myotonic dystrophy, there is a clear relation between adherence to treatment and survival (41–44). Thus, the best compromise between patient comfort and efficacy must be sought for. Pattern of use (fragmentation vs. continuous use) can be an indicator of discomfort and side-effects (leaks, pain or discomfort related to interface), co-morbidities (nycturia, use of diuretics) or symptomatic patient-ventilator asynchrony (Figure 1) (7, 45, 46). Evolution of total time spent under NIV and changes in pattern of use may be indicative of an exacerbation with a risk of hospital admission (47).

#### Leaks

Leaks in LTNIV are either intentional (i.e., related to exhalation valves to avoid rebreathing) or unintentional (i.e., caused for instance by a poorly adapted interface, too high insufflation pressures, or mouth leaks). Intentional leaks depend on the type of mask and insufflation pressures: interfaces have their predefined leak vs. pressure curves (48). In barometric modes, which are presently by far the most frequently used, ventilators adapt the airflow generated by the turbine to the intentional leak to ensure that the pressure attained in the airways reflects settings determined by the clinicians. Unintentional leaks are by definition unpredictable, most often variable, may differ between inspiratory and expiratory phases, and can be influenced by elements such as facial morphology, type of interface (facial *vs*. nasal mask), body position, sleep stages or ventilator settings. More importantly, they may interfere with the detection of patient's changes in inspiratory flow (which triggers pressurization or induces cycling). Leaks are thus the most frequent etiology of patient-ventilator asynchrony. They may induce significant and sometimes severe desaturations due to hypoventilation or to a “dilution effect” in patients with supplemental oxygen and can impact on quality of sleep (49). Also, leaks have a detrimental effect on reliability of data provided by ventilator software such as tidal volume, minute ventilation, or percentage of cycles triggered or cycled by the ventilator (3). This may be particularly problematic in automated modes with volume targeting or providing a continuous assessment of airway resistance.

Most built-in monitoring systems coupled to LTNIV ventilators provide data about leaks. However, leaks are estimated and reported differently according to manufacturers, which may misguide the clinician (i.e., average leaks, average leak only during expiratory phase, average leaks with or without intentional leaks) (3, 50, 51). Bench test studies have shown that reliability of reported leaks may vary considerably according to device used and to absolute value of leaks (3, 5, 52). Arbitrary threshold values for acceptable leaks are commonly reported by several manufacturers: the threshold of 24 L/min often referred to was first mentioned in a study by Teschler et al. (53): the relevance of this value is questionable today since the efficacy of ventilator turbines and their capacity to compensate for leaks has improved substantially. A statement by the French GAV-O_2_ group suggested that leaks are considered troublesome essentially 1/ if they impact on patient comfort and compliance and 2/ if they cause recurrent nocturnal desaturations and episodes of hypercapnia (46).

For the patient, the variability of leaks and peak levels even of short duration may be a more important source of discomfort than the mean or median leak level.

#### Tidal Volume and Minute Ventilation

Tidal volume (V_T_) and minute ventilation (MV) are important goals for the clinician when determining ventilator settings: a usual target for V_T_ is 8–10 ml/kg of ideal body weight. The accuracy of V_T_, and thus MV, depends on the reliability of ventilator software, on pressure values and on leaks (54, 55). In a bench study of 7 home ventilators, bias between measured and reported V_T_ ranged from 66 to 236 ml (3). This may be problematic in automated volume-targeted modes. A high variability of tidal volume may *per se* be suggestive of leaks.

#### Cycles Triggered or Cycled by the Device

**Figure 2:**
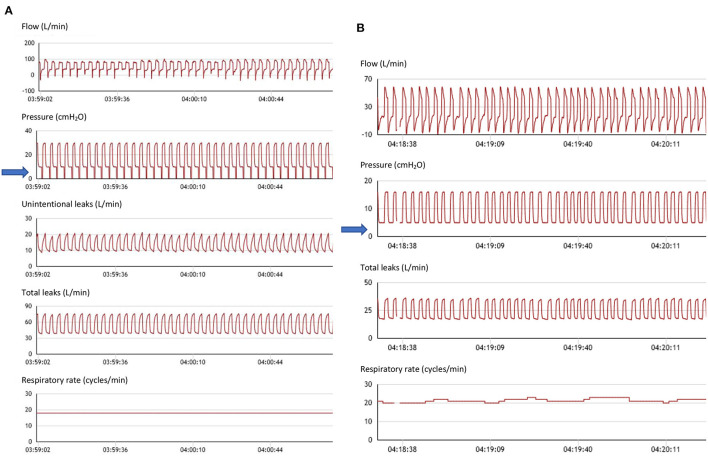
Spontaneous vs. controlled cycles. **(A)** From top to bottom: flow (including leaks), pressure, unintentional leaks (i.e.: difference between total leak flow and estimated flow through the leak valve (small holes) of the mask), total leaks, and respiratory rate. Seventy six-year-old female subject with kyphoscoliosis, obstructive sleep apnea and obesity; ventilator settings: ST mode (spontaneous/timed); IPAP (Inspiratory positive airway pressure): 30 cmH_2_O; EPAP: 10 cmH_2_O (Expiratory positive airway pressure); Back-up respiratory rate (BURR): 18 cycles/min. Nasal mask (facial mask not tolerated). Flow tracing shows intermittent flattening of inspiratory curve suggesting persisting flow limitation (upper airway collapse to be compensated by increasing EPAP). Blue arrow: pressure tracing shows vertical marks associated with each cycle indicating controlled cycle (i.e., delivered by ventilator). On this segment, patient is continuously on back-up respiratory rate. Low level of intentional leaks. **(B)** From top to bottom: flow (including leaks), pressure, total leaks, and respiratory rate. Fifty four-year-old male subject with restrictive disorder. Ventilator settings: ST mode (spontaneous/timed); IPAP (Inspiratory positive airway pressure): 16 cmH_2_O; EPAP: 5 cmH_2_O (Expiratory positive airway pressure); Back-up respiratory rate (BURR): 16 cyc/min; nasal mask. Blue arrow: as opposed to **(A)**, all cycles are triggered by the patient (i.e., spontaneous). Normal aspect of flow and pressure tracings. DirectView software, Philips Respironics.

Quantifying the percentage of cycles triggered by the patient is theoretically an important information. It allows to determine to what extent the patient's respiratory rate (RR) is captured by the ventilator, i.e., to what extent ventilatory support approaches a controlled mode (Figure 2). This may be a goal for clinicians in neuromuscular disorders for instance, to rest inspiratory muscles, or to decrease residual apneas in obesity hypoventilation (56). However, in the presence of leaks, and/or upper airway obstruction, a low percentage of triggered cycles may reflect the fact that the ventilator does not detect the patient's inspiratory efforts. Conversely, a high percentage of cycles triggered by the patient, i.e., a spontaneous mode, may be an indicator of patient-ventilatory synchronization. This parameter must therefore be interpreted with caution, especially in the presence of leaks.

Ventilators also provide the percentage of respiratory cycles cycled by the patient i.e., for which the predefined percentage of peak inspiratory flow is reached. A high percentage of respiratory cycles cycled by the patient may be considered as evidence of appropriate patient ventilator synchronization. Conversely, a high percentage of cycling by the device may reflect either a “controlled mode” or leaks with or without patient ventilator asynchrony.

#### Residual Respiratory Events Under LTNIV

**Figure 3:**
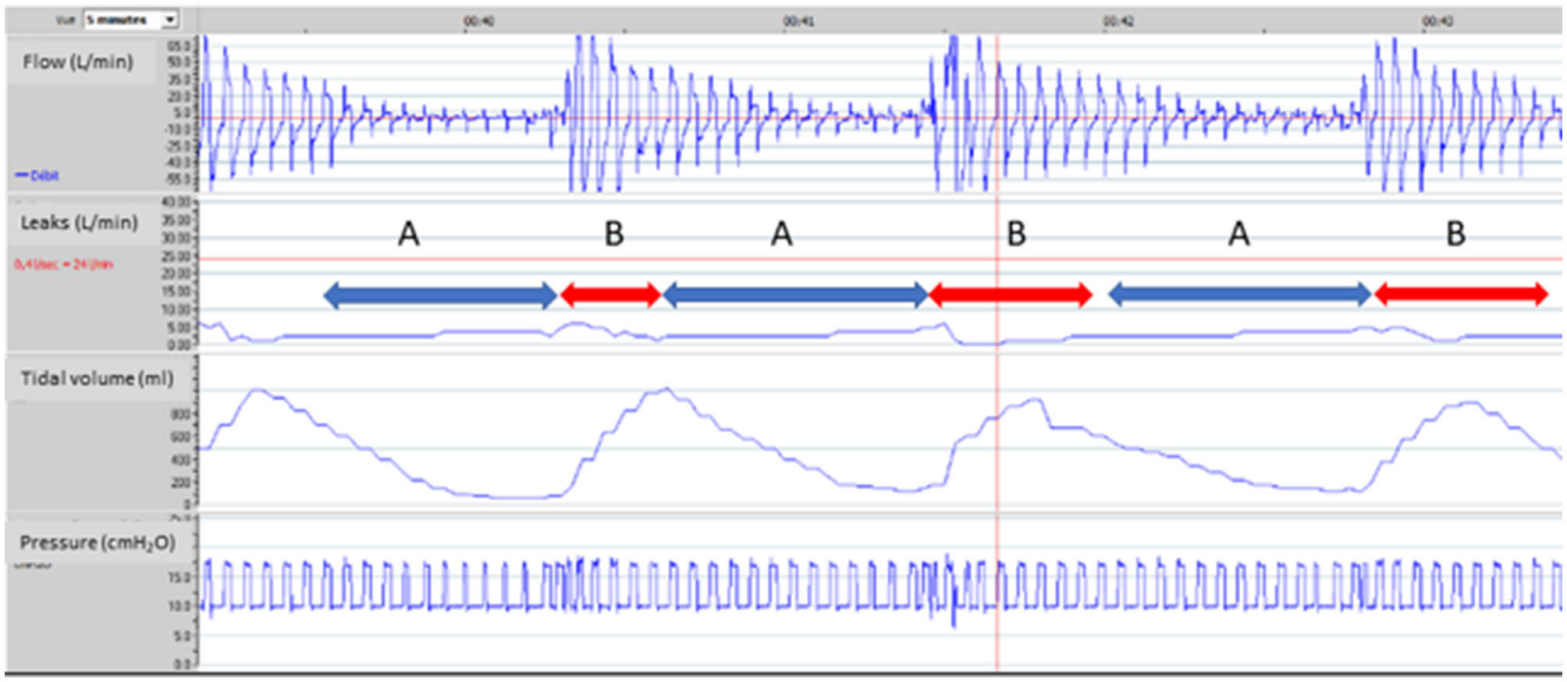
Upper airway obstruction under NIV. 65-year-old male subject, obesity-hypoventilation with obstructive sleep apnea syndrome (OSAS). Bi-level pressure support ventilator, ResScan software, ResMed. Facial mask. Ventilator settings: ST Mode (spontaneous/timed), IPAP (Inspiratory positive airway pressure): 20 cmH_2_O; EPAP (Expiratory positive airway pressure): 10 cmH_2_O; BURR (Back-up respiratory rate): 16 cycles/min. From top to bottom: flow, unintentional leaks (i.e.: difference between total leak flow and estimated flow through the leak valve of the mask), tidal volume, pressure. 5 min window. Red line on leaks tracing (24L/min) is a threshold value suggested by manufacturer for upper limit of acceptable leaks (see text for comments). Event **A**: marked decrease in flow with tracing suggesting increase in upper airway resistance (leading to intermittent complete obstruction); simultaneous decrease in tidal volume without increase in leaks. Event **B**: sudden transient resumption of flow with a simultaneous increase in tidal volume. Increase in upper airway resistance could be related to an insufficient “pneumatic splint” effect in spite of rather high insufflation pressures, or to glottic closure (further characterization would require respiratory polygraphy). In a patient with a known OSAS, a pragmatic trial of increasing EPAP is an option. Value of IPAP should be increased accordingly to maintain same level of pressure support (if tolerated). Use of a facial mask may also contribute to these events, and may be replaced by nasal mask with chin strap if tolerated.

**Figure 4:**
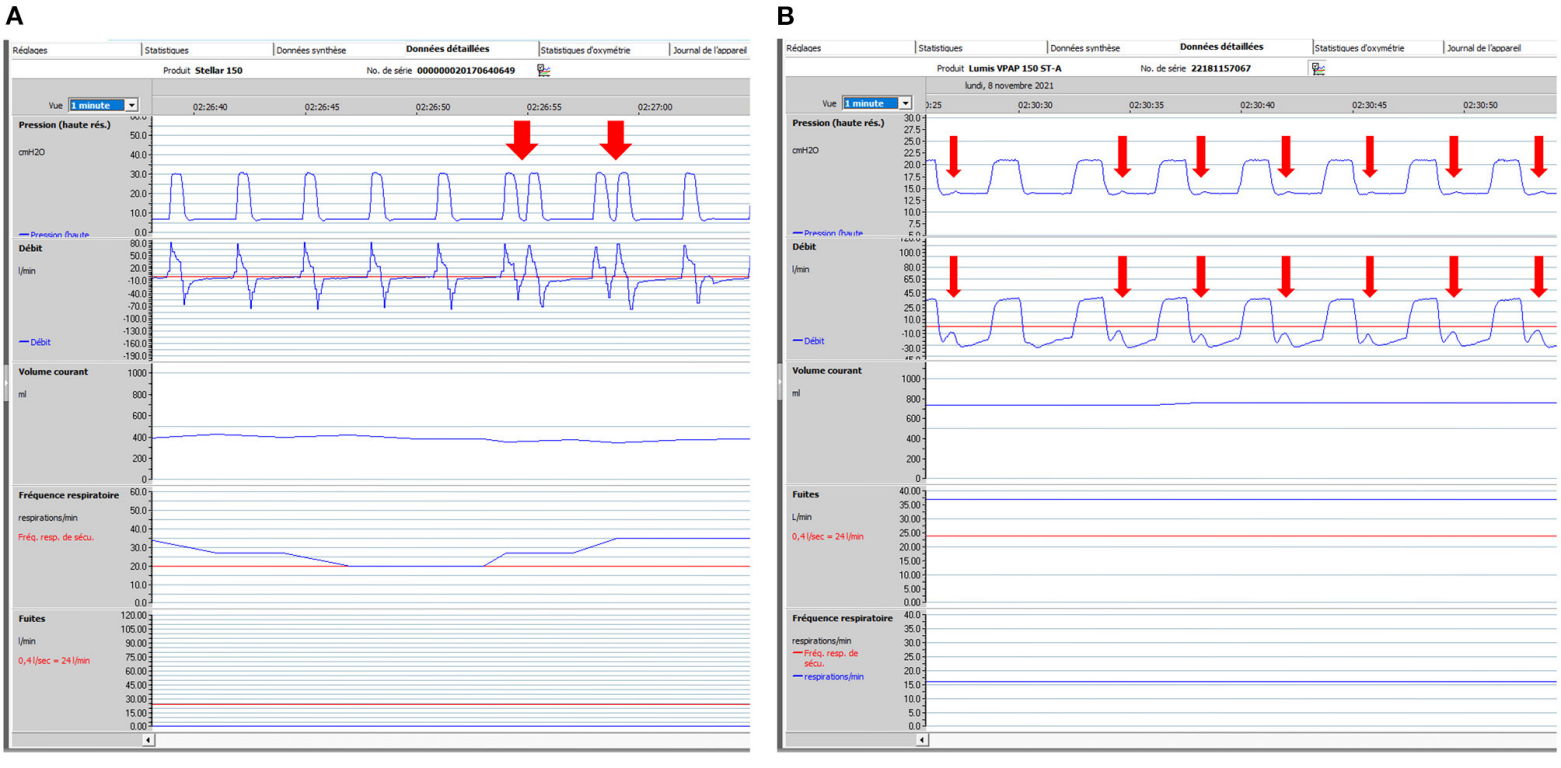
Patient ventilator asynchrony (PVA): illustrative ventilator tracings. One-minute windows. From top to bottom: pressure, flow, tidal volume, respiratory rate and unintentional leaks (i.e., without intentional leak through exhalation valve of mask). Rescan software, ResMed. **(A)** 74-year-old male subject with severe COPD (FEV1: 20% of predicted). Bi-level pressure support ventilator, Rescan software, Resmed. Ventilator settings: ST (spontaneous/timed) mode; IPAP (Inspiratory positive airway pressure): 30 cmH_2_O; EPAP (Expiratory positive airway pressure): 7 cmH_2_O; BURR (back-up respiratory rate) 20 cycles/min. Red arrows show intermittent double-triggering. In this case, leaks are not in cause. Possible causes are dysfunction of ventilator, too high inspiratory trigger sensitivity, too short minimal inspiratory time (TI_MIN_) with prolonged inspiratory efforts. Adjustments of settings (if required) to be considered are: to increase TI_MIN_; to adapt cycling sensitivity (at a lower percentage of peak inspiratory flow, which delays cycling); to increase IPAP and reduce rise time; or to decrease sensitivity of inspiratory trigger. **(B)** 67-year-old male subject with obesity hypoventilation and severe OSAS. Bi-level pressure support ventilator, Rescan software, Resmed. Ventilator settings: ST (spontaneous/timed) mode; IPAP (Inspiratory positive airway pressure): 21 cmH_2_O; EPAP (Expiratory positive airway pressure): 14 cmH_2_O; BURR (back-up respiratory rate) 16 cycles/min. Red arrows show low amplitude repeated increases in flow and pressure which represent unrewarded efforts (i.e., inspiratory efforts by the patient which do not trigger the ventilator). Among possible causes are: inappropriate setting of inspiratory trigger sensitivity, increase in upper airway resistance, leaks, intrinsic PEEP (Positive end expiratory pressure), decrease in inspiratory muscle function. For all PVA, control of leaks is mandatory before adjusting other settings.

**Figure 5:**
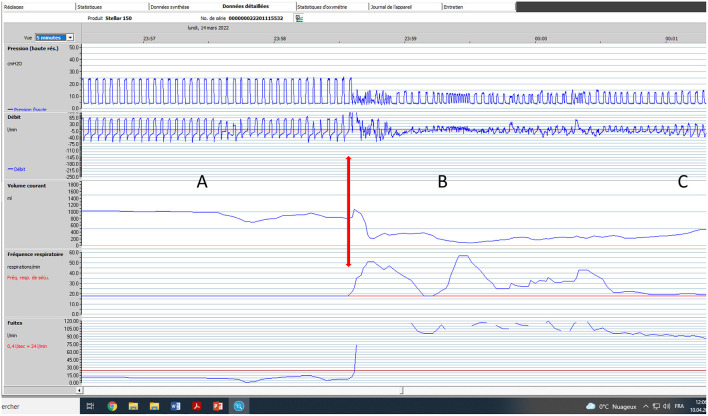
Impact of major leaks. Seventy five-year old male subject with severe COPD (GOLD D; FEV1: 17% of predicted). Bi-level pressure support ventilator, S/T mode (spontaneous/timed); IPAP (Inspiratory positive airway pressure): 24 cmH_2_O; EPAP (Expiratory positive airway pressure): 4 cmH_2_O; Back-up respiratory rate (BURR): 18 cycles/min. Facial mask. Five-minute window, Rescan software, ResMed. **(A)** normal tracing albeit for a few cycles with decrease in flow; **(B)** Vertical arrow marks appearance of major leaks (could be related to transient displacement of interface). Pressure tracing shows episodes of auto-triggering, and double triggering. Marked drop in pressure, flow and V_T_ (explained by magnitude of leaks). **(C)** As leaks progressively decrease, breathing pattern becomes more regular; pressure and flow increase progressively.

The presence of residual respiratory events is frequent and has a documented impact on quality of sleep and sleep structure (23, 57–60) (Figures 3–5). Severe patient-ventilator asynchrony may compromise the efficacy of NIV. Also, events such as recurrent upper airway obstruction may affect survival in ALS (9). It is thus important to evaluate, and correct these events.

Most devices report residual respiratory events occurring under NIV: they are labeled as apnea, hypopnea, and sometimes even classified as “central” or “obstructive” according to complex algorithms and estimation of upper airway resistance using the force oscillation technique, the shape of the flow curves or both. The relevance of these parameters in LTNIV is not well-established: these parameters are derived mainly from the use of CPAP in sleep-related disordered breathing (SRDB), and do not take into account the higher level of complexity of undesired residual (or *de novo*) respiratory events associated with LTNIV. Very few studies have assessed the reliability of these data. Georges et al. showed that the apnea-hypopnea index (AHI) reported by one specific device in patients with obesity-hypoventilation was well-correlated with a manually read polysomnographic assessment, and that a threshold value of 10/hour for AHI allowed to discriminate with a high sensitivity and specificity subjects who were correctly ventilated vs. those for whom adjustments were required (61). In a similar study, Alvarez et al. compared manually scored polygraphy with built-in software data in 26 patients with obesity-hypoventilation: automated analysis of residual events was well-correlated with polygraphy; however, manually scored tracings from ventilator software provided more consistent results than automated software (62). More recently Aarrestad et al. compared manually scored polygraphy and data from ventilator software in 67 subjects on LTNIV for restrictive disorders: a value of AHI above 7.2/hour reported by ventilator software had a sensitivity of 93% (95%CI: 68–100) and a specificity of 92% (95%CI: 81–98) for detecting patients with an AHI > 10/hour scored by polygraphy (34).

#### Detailed Analysis of Flow and Pressure Curves and Coupling With Pulse Oximetry and Leaks

Ventilator software provides the possibility of a detailed cycle-by-cycle analysis of pressure and flow curves, while simultaneously providing curves of estimated leaks and tidal volume. Oximetry can be added to most devices to complete this analysis. This allows the detection of residual obstructive events with or without persistence of respiratory drive (63), may show periodic breathing, and patient ventilator asynchronies (rate asynchrony such as multiple triggering, auto-triggering, ineffective efforts, or intracycle asynchronies such as underassistance, or overshoot) (49). Coupling the detailed analysis of ventilator curves with oximetry and leaks allows a better assessment of relevance and mechanisms involved, and provides a rationale to adapt ventilator settings. Illustrative cases of contribution of pressure and flow curves are shown in Figures 2–5.

#### Contribution of Respiratory Polygraphy or Polysomnography

**Figure 6:**
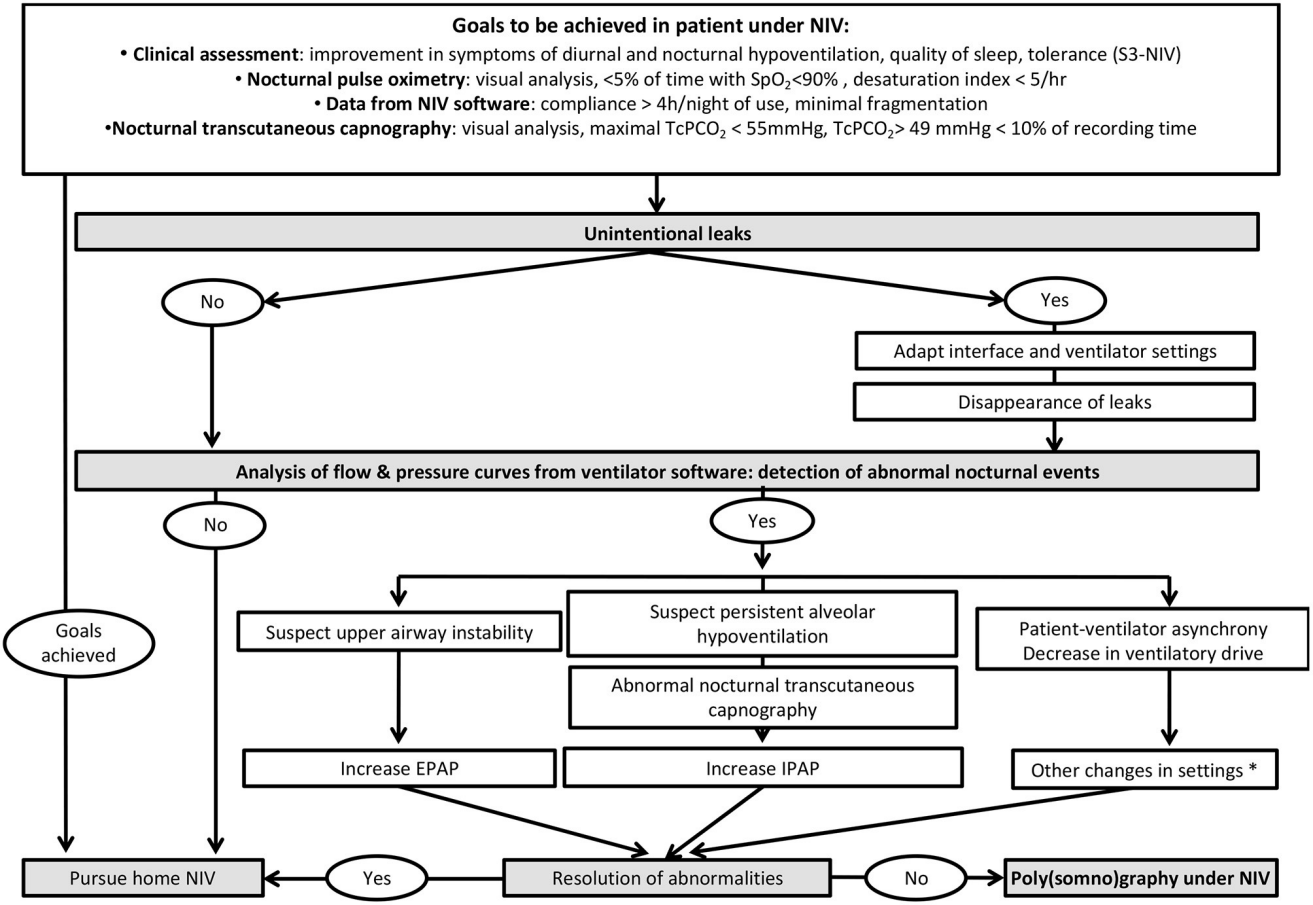
Proposed algorithm for a systematic approach to monitoring of patients under LNIV and analysis of ventilator software. *: the reader is referred to references 4 and 8 for further details.

Respiratory polygraphy (PG) or polysomnography (PSG), preferably coupled with TcPCO_2_, are considered as gold-standard for documenting undesired respiratory events occurring under LTNIV. These events have been reported by several studies (34, 45, 57, 58, 64, 65) and defined in detail by the SomnoNIV group (63). However, PG or PSG under NIV requires expertise. PSG is not easily available in many countries, and thus use of PSG for titrating and/or monitoring LTNIV, although recommended by the AASM (American Association of Sleep Medicine), is not standard practice (66). Some specialized centers use PSG for titration and follow-up (67). Proposed algorithms recommend PSG or PG only after a thorough analysis of symptoms, PtcCO_2_, ventilator software and correction of leaks (4, 68) (Figure 6).

#### Symptom Scores and Assessment of Health-Related Quality of Life (HRQL)

Specific HRQL questionnaires have been designed for patients under LTNIV, such as the SRI (Severe Respiratory Insufficiency Questionnaire) which is the most widely used in this setting, with 16 translations and an “App” version available (69, 70). This type of questionnaire is however mostly designed for clinical studies, and not for follow-up of individuals.

A disease-specific symptom score (S^3^-NIV) has been recently developed (71) and is undergoing several translations: it is an 11-item Lickert scale type questionnaire which can be auto-administered, and provides 2 sub-scores: “respiratory symptoms” and “sleep and NIV-related side effects.” This score is perfectly suitable for clinical practice. It can be coupled with simple tools for assessing mood disturbances such as the Hospital Anxiety and Depression scale (72). These tools do not replace however the necessary face-to-face exchange with the patient and/or his/her caregivers to appreciate the relevance of findings downloaded from ventilator software and mentioned in symptom scores such as the S3-NIV.

#### Strategies to Detect Ineffective Control of Nocturnal Hypoventilation by NIV

NIV should be systematically monitored (4, 46). As mentioned previously, residual nocturnal hypoventilation, unintentional leaks, patient-ventilator asynchrony or abnormal respiratory events are frequent under NIV and have a negative impact on patient-related outcomes such as symptoms, HRQL and survival. It has been estimated that approximately one third of ventilated patients with normal daytime ABG and nocturnal SpO_2_ had residual nocturnal hypoventilation under NIV (28, 35, 73). Withholding from performing regular testing of NIV efficacy could therefore be detrimental.

Optimal modalities for monitoring of long-term ventilated patients remain a matter of debate. As previously mentioned, complete polysomnography (PSG) under NIV is performed by some groups, but is not feasible in most centers on a routine basis (74).

Alternative tools (such as oximetry, TcPCO_2_, or ventilator software) can be used alone or in combination. A step-by-step strategy starting by ABG and nocturnal SpO_2_ has been proposed by the SomnoNIV group (4) (Figure 6).

Over the past years, the use of TcPCO_2_ has been simplified (less frequent changes of probes, improved software and estimation of drift). Failure to retrieve data is rare (75) and instrumental drift of TcPCO_2_ is a minor problem when used by an experienced team (27). Several studies have shown that continuous TcPCO_2_ recording provides an accurate picture of the overnight time course of PaCO_2_ in CHRF under NIV. Experts propose different thresholds to define significant nocturnal hypercapnia: maximal TcPCO_2_ >49 mmHg, TcPCO_2_ >49 mmHg for >10% of recording time, TcPCO_2_ >55 mmHg for ≥10 min or an increase in TcPCO_2_ ≥10 mmHg above awake supine value to a value exceeding 50 mmHg for ≥10 min (33). The choice of a clinically relevant threshold may be influenced by 1/the bias between arterial and transcutaneous PCO_2_ according to the device used, 2/the etiology of the underlying chronic respiratory failure and 3/PaCO_2_ levels when NIV is started. Beyond this debate, capnography is currently a well-accepted surrogate procedure for diagnosing nocturnal residual hypoventilation in ventilated patients. Simultaneous recording of SpO_2_ with the same ear probe improves the clinical contribution of nocturnal transcutaneous capnography. Indeed, sampling rate and averaging of SpO_2_ and TcPCO_2_ recordings are different. Therefore, SpO_2_ traces can detect brief desaturations related to short ventilatory events while TcPCO_2_ traces allow evaluating overnight trends because of a longer lag time (4).

Significant leaks and/or abnormal residual respiratory events (i.e., flow reduction or patient-ventilator asynchronism) are frequently detected in patients with normal nocturnal TcPCO_2_ and SpO_2_ (23, 59, 64).

In a study comparing different tools and their combination, Georges et al. demonstrated that combining the signals provided by TcPCO_2_ and data from ventilator software provides the best noninvasive assessment of NIV efficacy (73). This approach allows monitoring of LTNIV at the hospital or at home without complex logistics. Interpretation of the results is simple and further analysis of detailed raw data provided by ventilator software can help clarify the underlying mechanism to optimize NIV settings, thus limiting the use of PSG to more complex cases.

A suggested strategy for nocturnal NIV monitoring and parameter optimization is summarized in Figure 6.

#### Patient-Ventilator Asynchrony and Its' Relevance

Several studies have reported the—sometimes frequent—occurrence of patient ventilator asynchrony (PVA) in patients under LTNIV (23, 57–59, 63, 64, 76) (Figures 4, 5). Their specific semiology and a suggested framework for their analysis has been proposed by the SomnoNIV group (49). Briefly, after correction of leaks and obstructions, which are the major contributors to PVA, a visual analysis of tracings is necessary to detect the presence of rate asynchrony and/or intracycle asynchrony. Rate asynchrony is further subdivided into multiple triggering, uncoupling and ineffective efforts, while intracycle asynchrony can reveal either flow asynchrony (under-assistance) or phase asynchrony (premature or delayed cycling). Clinical relevance of PVA is still a matter of debate, especially if not associated with discomfort and hypoventilation and/or nocturnal desaturation. Scoring of PVA is not yet standardized in the medical literature, and prevalence can vary considerably according to working definitions (23). Seeking for PVA seems however appropriate after correction of leaks in the presence of symptoms and/or unexplained desaturations.

#### Tele-Monitoring

Although the use of tele-monitoring has become in many countries standard practice for following patients treated by CPAP and Sleep-related breathing disorders (SRBD), and, in some countries, is even required for its reimbursement, this is not the case for LTNIV. Many devices used for LTNIV have the necessary technology built-in, but each manufacturer has its software, and way of reporting certain parameters, representing a challenge for clinicians. Reliability of data remains an issue for some data, as previously discussed (77). In spite of these limitations, home titration and follow-up of LTNIV are emerging strategies. Use of tele-monitoring as an adjunct to home initiation of LTNIV is also an option, and has been shown to be cost-effective in selected indications such as CHRF related to neuromuscular disorders or other restrictive lung diseases (78, 79). Several studies suggest a benefit on healthcare utilization (emergency visits, hospital admissions) and cost-effectiveness of tele-monitoring in ALS (80–82). Preliminary studies have explored the possibility of early detection of exacerbations of COPD under LTNIV (47). For instance, changes in number of hours of NIV use may be a sign of imminent exacerbation, of worsening of underlying disease, or of discomfort, and can be a useful signal for early home intervention (7, 47). On-going trials (reviewed by Borel et al.) (77) will help clarify the modalities of a cost-effective use of tele-monitoring in NIV, and importantly, its benefit (or absence of) on relevant outcomes (such as HRQL, survival, hospital admissions). Points of potential interest for telemonitoring are: follow-up after initiation of NIV following an acute episode of respiratory failure (effective daily use, leaks etc.), progressive titration of NIV after home initiation of LTNIV, and, once specific algorithms will have been validated, early detection of exacerbations or deterioration of disorder causing CRF. The present tools for tele-monitoring already provide a limited “traffic light system” (red, yellow and green icons) usually based on leaks, AHI and compliance. A more elaborate signaling system, using limits for tidal volume, minute ventilation, respiratory rate etc. has yet to be validated in clinical practice, the risk being an unwarranted increase of “red lights.” The possibility of adapting certain parameters at a distance (pressure support, expiratory positive airway pressure, or back-up respiratory rate) allows progressive changes in these settings for increased patient comfort and tolerance, and reduces the requirement for in-hospital titration (83).

#### The Contribution of “Big Data”

The concept of “Big data” refers to access to a massive quantity of data, its speed of acquisition, and the diversity and heterogeneity of data sources, with potential uncertainties regarding data quality (84). The widespread use of telemonitoring for CPAP has opened the way to a new approach for exploring patient pathways, identifying potentially relevant phenotypes, and elements impacting on adherence and efficacy. For instance, Liu et al. provided very useful data for a better understanding of trajectories of patients with central sleep apnea (emergent, transient, persistent) after initiation of CPAP (85). Technical information such as the impact of changes of interface can be easily assessed through daily longitudinal follow-up data at a large scale. Detailed analysis of day by day use of devices reveals behavioral patterns which improve our understanding of how patients adhere and accept their treatment (86, 87). Recently, it has been possible through telemonitoring to evaluate the impact of the lockdown during the COVID pandemic on adherence to CPAP or NIV (88, 89). Undoubtedly, in a near future, this type of information will become increasingly available and provide a useful adjunct to clinical trials.

## Conclusions

The relevance and necessity of a regular monitoring of patients under LTNIV is clearly established, and has an impact on prognosis and HRQL. The progressive shift from hospital-based to home-based assessment over the past years is important for patient comfort and cost-effectiveness of LTNIV. Strategies for monitoring LTNIV include clinical assessment, symptom scores, simple tools such as TcPCO_2_ or oximetry, ventilator software, and in specific situations, respiratory polygraphy or polysomnography. Although telemonitoring of patients treated by CPAP for SRBD is widely accepted, its use in LTNIV still has to be explored and shown to be cost-effective. Access to big data may provide additional information to better clarify phenotypes of responders and non-responders to LTNIV.

## Author Contributions

J-PJ, CC, PP, MG, and CR have contributed to the writing, designing, and revision of the final version of this manuscript and are accountable for the content of this manuscript. All authors contributed to the article and approved the submitted version.

## Conflict of Interest

The authors declare that the research was conducted in the absence of any commercial or financial relationships that could be construed as a potential conflict of interest.

## Publisher's Note

All claims expressed in this article are solely those of the authors and do not necessarily represent those of their affiliated organizations, or those of the publisher, the editors and the reviewers. Any product that may be evaluated in this article, or claim that may be made by its manufacturer, is not guaranteed or endorsed by the publisher.
